# Ketoprofen Lysine Salt vs. Ketoprofen Acid: Assessing the Evidence for Enhanced Safety and Efficacy

**DOI:** 10.3390/life15040659

**Published:** 2025-04-16

**Authors:** Agnese Graziosi, Michele Senatore, Gianluca Gazzaniga, Stefano Agliardi, Arianna Pani, Francesco Scaglione

**Affiliations:** 1 Department of Oncology and Hemato-Oncology, University of Milan, 20122 Milan, Italy; 2 Chemical-Clinical Analysis Unit, ASST Grande Ospedale Metropolitano Niguarda, 20162 Milan, Italy; 3 Department of General Surgery and Surgical Specialty Paride Stefanini, Sapienza University of Rome, 00185 Rome, Italy; 4 Niguarda Cancer Center, ASST Grande Ospedale Metropolitano Niguarda, 20162 Milan, Italy

**Keywords:** ketoprofen acid, NSAID, ketoprofen lysine salt, PK, PD

## Abstract

Endoscopic investigations reveal that a significant majority of individuals taking NSAIDs exhibit acute hemorrhages and mucosal erosions within the gastroduodenal lining. Ketoprofen acid (KA) is a potent NSAID with established efficacy and cardiovascular tolerability, but its gastric tolerability is a recognized limitation. To mitigate this, ketoprofen lysine salt (KLS) was developed. This review evaluates the pharmacological advantages of KLS over KA. While both KA and KLS maintain similar potency, KLS offers distinct advantages. Firstly, KLS demonstrates superior gastrointestinal protection through enhanced antioxidant properties and upregulation of mucosal defenses, as evidenced by both in vitro and in vivo studies. Secondly, KLS exhibits significantly faster absorption, leading to a more rapid onset of analgesic effects; this is attributed to its increased solubility and faster achievement of therapeutic concentrations. In essence, KLS addresses the gastric tolerability issues of KA while providing a quicker onset of action, making it a valuable alternative for patients requiring NSAID therapy, particularly those with gastric sensitivities or in need of rapid pain relief.

## 1. Introduction

Developed in 1967 and approved in Europe for clinical use in 1973, ketoprofen, a potent non-steroidal anti-inflammatory drug (NSAID) of the arylpropionic acid class, has been widely used as ketoprofen acid (KA) because it effectively reduces inflammation, pain, and fever [1]. NSAIDs, as a drug class, play a vital role in managing pain and inflammation across various conditions. Inflammation, a multifaceted biological reaction triggered by cellular damage or infection, underlies a diverse array of pathological conditions, including malignancies, Alzheimer’s disease, type 2 diabetes mellitus, rheumatoid arthritis, and asthma [2]. While the acute inflammatory response plays a crucial role in the body’s defense mechanisms, chronic inflammation can lead to tissue damage and contribute to the pathogenesis of numerous diseases [3]. NSAIDs have a long history of use in managing inflammation [4,5]. These agents typically exert their effects by inhibiting cyclooxygenase (COX), an enzyme that catalyzes the production of prostaglandins (PGs), potent mediators of inflammation [6]. However, conventional NSAIDs non-selectively inhibit both COX-1 and COX-2 isoforms, a characteristic associated with significant gastrointestinal side effects such as ulceration and an elevated risk of hemorrhage [7].

Ketoprofen, in particular, is valued for its quick absorption, straightforward metabolic pathway, efficient penetration of the blood–brain barrier, and significant pain-relieving capabilities [8]. However, while KA demonstrates favorable efficacy, cardiovascular, and renal tolerability, its use is limited by the risk of gastrointestinal adverse reactions [9,10]. The acidic nature of KA contributes to gastric irritation, increasing the likelihood of ulcers, bleeding, and other complications. This limitation has driven research into new ketoprofen-based compounds with improved gastric profiles.

Ketoprofen exhibits analgesic, antipyretic, and anti-inflammatory properties through non-specific COX-1 and COX-2 inhibition, thought to occur mainly in peripheral sites, although recent studies indicate that ketoprofen also appears to have central effects [11]. The mechanisms underlying NSAID-induced gastrointestinal injury are a complex, multifactorial phenomenon involving both local and systemic effects [12]. The prostaglandin-dependent mechanism of NSAID-induced gastrotoxicity has been extensively studied [4]. NSAIDs inhibit cyclooxygenase (COX) enzymes, which are responsible for the synthesis of prostaglandins. Prostaglandins are hormone-like substances that play a key role in the inflammatory response, pain perception, and fever. There are two main isoforms of COX: COX-1 and COX-2. COX-1 is constitutively expressed in most tissues and is involved in maintaining normal physiological functions, such as gastric mucosal protection and platelet aggregation. COX-2 is inducible and is primarily expressed at sites of inflammation. Traditional NSAIDs, like ketoprofen, non-selectively inhibit both COX-1 and COX-2. The inhibition of COX-2 leads to the reduction in prostaglandins, resulting in the desired anti-inflammatory, analgesic, and antipyretic effects. However, the inhibition of COX-1 can disrupt protective mechanisms in the gastrointestinal tract, leading to an increased risk of ulcers, bleeding, and other complications [4]. More recent research has highlighted the implication of prostaglandin-independent pathways in the pathogenesis of Upper Gastrointestinal Bleeding (UGIB) induced by individual NSAIDs [13]. Topical effects are thought to initiate gastric erosions and have been linked to the common properties of NSAID accumulation in gastric epithelial cells and uncoupling of mitochondrial oxidative phosphorylation and electron transport [14]. NSAIDs have been shown to increase the activity of endothelin-converting enzyme, thereby suppressing constitutive nitric oxide synthase (eNOS) and endothelial nitric oxide, a key mediator in maintaining gastric mucosal integrity [8]. Similarly, the observed inhibition of COX-independent cystathionine-γ-lyase (CSE) expression in the gastric mucosa was associated with increased levels of hydrogen sulfide, another important anti-apoptotic and anti-anti-necrotic mediator in the gastrointestinal tract [8]. NSAIDs may also activate repair/adaptive mechanisms by stimulating the innate ability of the gastric mucosa to counteract damage associated with various insults; these activities may both stimulate healing and the development of resistance mechanisms to insults [8]. These COX-independent mechanisms may result in increased growth factor expression, mucin production, and modulation of gastric secretion that counteract the damage resulting from prostaglandin inhibition. In addition to COX inhibition, NSAIDs may exert other effects that contribute to their therapeutic actions. Some NSAIDs have been shown to modulate the production of other inflammatory mediators, such as cytokines and chemokines. They may also affect neutrophil function, inhibit the production of free radicals, and influence cell signaling pathways [9,14]. 

Several approaches have been explored to mitigate the gastrointestinal limitations of KA, including modified delivery systems (e.g., hydrogels, proliposomal powders), prodrug and codrug formulations, and novel chemical entities (e.g., ATB-352, zinc complexes). Among these, ketoprofen lysine salt (KLS) stands out due to its demonstrated improvements in gastric tolerability and its unique pharmacokinetic profile, contributing to a rapid onset of action in treating pain and fever [15,16]. KLS, formed by combining ketoprofen with the amino acid L-lysine, offers a potential solution to the gastrointestinal issues associated with KA. The addition of L-lysine enhances the water solubility of ketoprofen, which may lead to faster dissolution and absorption and also seems to exert a protective effect on the gastric mucosa.

However, gaps remain in the comprehensive understanding of the comparative benefits and drawbacks of KLS versus KA. Specifically, there is a need for a detailed review that synthesizes the available evidence on their efficacy, tolerability, and pharmacokinetic profiles to inform clinical decision-making. A thorough examination of the evidence is crucial for clinicians to weigh the potential advantages of KLS, such as reduced gastrointestinal toxicity and more rapid pain relief, against any potential disadvantages or uncertainties. Furthermore, researchers need a clear understanding of the current state of knowledge to identify areas where further investigation is warranted.

This review aims to critically evaluate the advanced features of KLS compared to KA. It will focus on preclinical and clinical evidence related to efficacy, tolerability, and pharmacokinetics. By synthesizing the current evidence, this review seeks to inform clinicians, researchers, and patients about the potential advantages and disadvantages of KLS compared to KA, ultimately contributing to improved clinical decision-making and patient care.

## 2. Materials and Methods

This paper reviews the current evidence supporting the difference in ketoprofen acid versus ketoprofen lysine salt for pain management and antipyresis. For this review, the available literature was searched using PUBMED and EMBASE databases using the search terms (ketoprofen OR ketoprofen acid) AND (ketoprofen lysine salt OR KLS). Results were limited to English-language publications. Duplicate records were removed. Specific publications included in this review were then selected manually from the search results for their relevance to this topic. Specifically, we selected papers (a) comparing pharmacokinetic profiles of KLS and KA, with a focus on absorption rates and time to peak plasma concentrations; (b) assessing the evidence for improved gastrointestinal tolerability with KLS, including both preclinical and clinical studies; (c) evaluating the analgesic and anti-inflammatory efficacy of KLS in comparison to KA; (d) summarizing the available data on the safety and tolerability of both KLS and KA, including potential adverse effects beyond the gastrointestinal system.

## 3. Discussion of Findings

### 3.1. Ketoprofen

From the first studies, the drug proved to be very powerful. In several experimental models (rats, mice, rabbits, guinea pigs, and pigeons) ketoprofen displayed potent activity against acute, subacute, and chronic inflammation [1]. These tests showed ketoprofen to be 20 times more potent than ibuprofen, 80 times more potent than phenylbutazone, and 160 times more potent than acetyl salicylic acid in reducing inflammation from carrageenin-induced abscesses in rats. The greater potency of ketoprofen compared to ibuprofen and diclofenac has also been demonstrated in clinical studies [11,17]. The drug’s potency was generally equivalent to that of indomethacin in most models. However, indomethacin shows significantly higher toxicological effects than ketoprofen. The acute oral LD50 of Indomethacin base for rats was 12 mg/kg, while the acute oral LD50 of ketoprofen for rats was 64 mg/kg [18]. 

Extensive testing in the United States, confirming foreign clinical experience, demonstrated that ketoprofen is effective in the treatment of arthritis. Furthermore, the drug has a well-defined safety profile that offers significant advantages over other NSAIDs in controlled studies [19]. United States approval of clinical use of ketoprofen in osteoarthritis and rheumatoid arthritis was granted in January 1986.

The clinical pharmacology of ketoprofen is well described. Briefly, in adults (healthy volunteers and patients), ketoprofen administered as a regular-release oral tablet at doses of 50–200 mg is rapidly absorbed, with a bioavailability of ≥92%. The peak plasma concentrations (Cmax) of 2.6–23.0 mg/L occur between 0.8 and 2.4 h after a dose (tmax). Ketoprofen is extensively metabolized to unstable acyl glucuronide conjugates; minimal quantities of unchanged drug are eliminated in the urine and bile, regardless of the patient’s age or renal function. Ketoprofen is widely distributed, including within the central nervous system (CNS), and the terminal elimination t½ is 1.5–2 h for the oral formulation, 2.2 h for rectal administration, and 2 h for intravenous administration.

Alongside its high potency, ketoprofen shows high cardiovascular safety, which places it as one of the most tolerable NSAIDs at this level. The risk of cardiovascular (CV) complications has been associated with NSAIDs particularly after the introduction of COXIBs and the subsequent withdrawal of rofecoxib [20]. The proposed mechanism by which NSAIDs lead to an increase in cardiovascular events, such as myocardial ischemia and stroke, is related to the selective action of coxibs on COX-2, which is associated with reduced prostaglandin I2 (PGI2 or prostacyclin) production that stimulates vasodilation and inhibits platelet aggregation. On the other hand, the failure to inhibit COX-1 increase the production of thromboxanes that promote thrombosis and blood vessels constriction [21]. However, this theory of balanced versus unbalanced COX inhibition is debated because non-selective NSAIDs have also been associated with increased cardiovascular risk [22]. Myocardial failure was associated with Etoricoxib, lumiracoxib, and rofecoxib, as well as diclofenac and ibuprofen [23,24]. These findings suggest that mechanisms other than COX-2/COX-1 selectivity may be implicated in cardiovascular toxicity. Interestingly, a recent study reports that diclofenac and ketoprofen are both able to induce an increase in ROS production, mitochondrial membrane potential loss, and proteasome modulation in immortalized human cardiomyocytes, but only diclofenac significantly disrupted these intracellular processes, leading to cell death. Notably, diclofenac diminished the proteasome 26S DC complex, a phenomenon potentially linked to intracellular oxidized protein accumulation. These data suggest that ketoprofen exposure elicits a manageable stress response in cardiomyocytes, whereas diclofenac induces cell death [25]. A large epidemiological study to assess individual NSAID-associated acute myocardial infarction (AMI) risk confirms that. A nested case–control study (8.5 million new users, 79,553 AMI cases, 1999–2011) was conducted using six databases. Adjusted odds ratios (OR) compared current to past use, pooled by one- and two-stage methods. Ketorolac showed the highest risk (OR 2.06), followed by indomethacin, etoricoxib, rofecoxib, diclofenac, etc., as shown in Table 1 [26]. 

However, in addition to its characteristics of potency and tolerability, KA, has a free acidic group in its chemical structure limits its appeal due to the risk of gastrointestinal adverse reactions.

Studies on more than 20,000 adults taking KA 200 mg once daily showed that around 13.02–13.5% had gastrointestinal symptoms [9]. The relative risks of upper gastrointestinal complications associated with the use of individual NSAIDs was estimated for KA to be 3.9, similar to nimesulide (3.8), diclofenac (3.3), and naproxene (4.1) [10]. 

To preserve the important characteristics of efficacy and general tolerability of KA, numerous studies have been undertaken to find a pharmaceutical form of ketoprofen that was more tolerable at the gastroduodenal level than both KA and other NSAIDs. The aim was to develop derivatives that would be ideally completely safe for the stomach and cause as few other side effects as possible. The analgesic, antipyretic, and anti-inflammatory effects of KA should remain unchanged. All these new compounds have recently been comprehensively reviewed by Kuczyńska and Nieradko-Iwanicka [12].

In our opinion, ketoprofen lysine salt (KLS) represents the most interesting compound among all new compounds derived from KA.

### 3.2. Ketoprofen Lysine Salt

**Figure 1:**
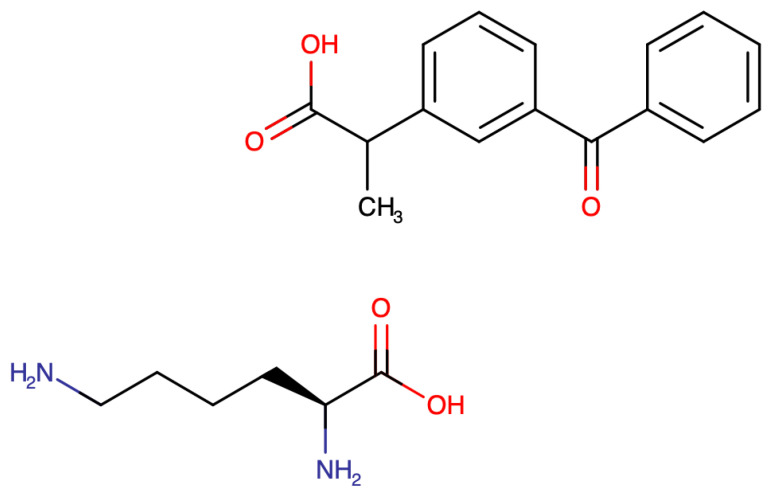
Ketoprofen lysine salt structure.

Dompè laboratories in Italy developed ketoprofen lysine salt (KLS) in the late 1970s (Figure 1). While KLS maintains the same potency as ketoprofen acid (KA), it exhibits improved tolerability, faster onset of action, and enhanced gastrointestinal safety, demonstrating a reduced risk of gastrolesivity [27]. 

The addition of lysine, on the other hand, is absolutely safe. Many studies have shown that lysine has no adverse effects up to 6 g per day, even for long-term intake. This limit is not remotely reachable with the intake of KLS even at the maximum permitted doses [28].

As previously said in the introduction, mechanisms underlying NSAID-induced gastrointestinal injury are complex, and the prostaglandin-dependent mechanism has been extensively studied. However, more recent research has highlighted the implication of prostaglandin-independent pathways in the pathogenesis of Upper Gastrointestinal Bleeding (UGIB) induced by individual NSAIDs. Topical effects are thought to initiate gastric erosions and have been linked to the common properties of NSAID accumulation in gastric epithelial cells and uncoupling of mitochondrial oxidative phosphorylation and electron transport [14]. NSAIDs have been shown to increase the activity of endothelin-converting enzyme, thereby suppressing constitutive nitric oxide synthase (eNOS) and endothelial nitric oxide, a key mediator in maintaining gastric mucosal integrity. Similarly, the observed inhibition of COX-independent cystathionine-γ-lyase (CSE) expression in the gastric mucosa was associated with increased levels of hydrogen sulfide, another important anti-apoptotic and anti-anti-necrotic mediator in the gastrointestinal tract. NSAIDs may also activate repair/adaptive mechanisms by stimulating the innate ability of the gastric mucosa to counteract damage associated with various insults; these activities may both stimulate healing and the development of resistance mechanisms to insults. These COX-independent mechanisms may result in increased growth factor expression, mucin production, and modulation of gastric secretion that counteract the damage resulting from prostaglandin inhibition [16]. 

Two mechanistic studies, using a well-established model of gastric mucosa injury, demonstrated the gastroprotective effect of KLS mucosa integrity. In the first study, gastric cells exposed to 6% ethanol, were incubated with KA or KLS or lysine for 24, 48, or 72 h. Cells treated with ethanol and KA appeared severely damaged with evident loss of integrity, while cells treated with ethanol and KLS appeared preserved by ethanol injury [16]. In a series of experiments, the authors demonstrated that L-lysine protects the gastric mucosa against NSAID-induced damage. In these experiments, ethanol was used as a gastric damaging agent, inducing the formation of reactive oxygen species (ROS) and subsequent lipid peroxidation, as evidenced by increased malondialdehyde (MDA) and 4-hydroxy-2-nonenal (4-HNE) levels. While KA failed to attenuate the ethanol-induced increase in these lipid peroxidation products, KLS significantly reduced MDA and 4-HNE formation [29]. This effect was also observed with L-lysine alone [29], suggesting that the lysine component of KLS exerts a direct antioxidant effect, potentially by scavenging ROS and preventing their interaction with membrane lipids, thereby preserving membrane integrity. KA was unable to modify the lipid peroxidation products, while KLS counteracted the increase in MDA and 4-HNE protein adducts due to ethanol treatment. The same happens using L-lysine, suggesting that gastric protection is due entirely to a marked antioxidant effect of L-lysine. However, another experiment in the same study highlights that KLS may protect the gastric mucosa through mechanisms other than those induced by L-lysine alone. Although L-lysine alone increased basal heme oxygenase-1 (HO-1) expression, it did not attenuate the HO-1 suppression induced by ethanol [30]. Conversely, KLS administration in the ethanol-challenged model elicited a marked, time-dependent increase in HO-1. This effect was also, to a lesser extent, observed with KA, indicating a potential dual role for ketoprofen on the gastric mucosa. While COX inhibition may contribute to mucosal damage, ketoprofen also appears to activate gastroprotective pathways, as evidenced by HO-1 upregulation. The salification of KA with lysine in KLS appears to potentiate this protective effect, likely through a synergistic interaction between L-lysine and ketoprofen, resulting in improved HO-1 expression and reduced ethanol-induced injury.

A second study investigated the effect of KLS on gastric mucosa maintenance and adaptive mechanisms evaluating some COX-independent mechanisms that may explain the specific pathogenic effects of individual NSAIDs on gastric mucosa [15]. 

KLS was compared to KA and ibuprofen (IBU). At the morphological level, KLS determined an evident protection of the epithelium from alcohol-induced damage that was not observed with KA, while IBU even worsened ethanol effects. The study was completed evaluating gene expression profiles of genes involved in the maintenance of gastric mucosa barrier integrity such as:

- MUC5B, a highly glycosylated component of mucus, forms a physical barrier that protects the epithelium from acid and pepsin [31];
- GALNT8, involved in mucin glycosylation, is essential for the proper formation and function of the mucus layer [32];
- CCK regulates gastric acid secretion and exerts protective effects on the gastric epithelium against damage from agents like ethanol [33];
- CLDN5, a protein of tight junctions, strengthens the intercellular connections, preventing the paracellular passage of damaging substances [34].

Interestingly, KA produced an upregulation on all genes but to a lesser extent than KLS, while IBU down-regulated CCK, upregulated MUC5B, and showed no effect on other genes. It is therefore interesting to note the different behavior between the two NSAIDs, with IBU being significantly more harmful than ketoprofen on the gastric mucosa monolayer. These results do not reflect the relative potency of the two drugs as COX inhibitors, thus suggesting that ketoprofen may stimulate protective/adaptive pathways on the gastric mucosa via a COX-independent mechanism.

In contrast with the above-mentioned studies supporting the beneficial protective effects of L-lysine on gastric mucosa, a recent study indicated that KLS had no gastroprotective effects in a rat model of ethyl alcohol intoxication and did not protect gastric mucosa from ethyl alcohol-induced damage [35]. Unfortunately, these data come from an experimental model that is very far from the anatomy and physiology of the human stomach. The rodent stomach is divided into a glandular portion, which has a thick wall covered by columnar epithelia, and a non-glandular one, generally thin-walled and lined with keratinized stratified squamous epithelium [36]. The human stomach is of the glandular type and is physiologically and anatomically more similar to that of large animals, such as dogs or monkeys, for example [37]. Dogs, in particular, have been most extensively used in translational gastrointestinal research because of the high similarity between canine and human gastrointestinal anatomy and physiology [38]. After this clarification, it is useful to report a study that documents the translatable nature of the previous in vitro studies to in vivo models. In vivo tolerability/toxicity studies were conducted comparing the safety profiles of KLS and KA, focusing on the evaluation of the gastrointestinal and renal tolerability of the drugs administered orally to dogs [39]. 

Macropathology and histologic analyses showed that none of the dogs in the KLS group had ulcers and/or erosions in the stomach. In contrast, two out of four dogs in the KA group displayed ulcers and/or erosions, with one of the two presenting a severe ulceration of the stomach, associated with subacute inflammation and congestion of the gastric mucosa. Interestingly, histologic analysis of kidneys suggested an increased tolerability of KLS also in this organ. These results demonstrate that KLS has a better gastrointestinal tolerability in vivo than KA. They also show, for the first time, that KLS has a better renal tolerability in vivo than KA, thus supporting the concept that L-lysine can counteract gastrointestinal and renal damage mediated by NSAID-induced oxidative stress.

All these studies taken together demonstrate that KLS has a lower gastric damage potential than KA and IBU. However, they cannot be considered conclusive; comparative clinical studies in humans are needed to draw conclusive data.

**Figure 2:**
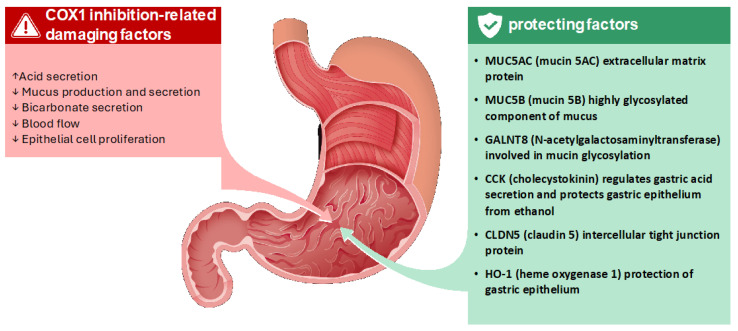
Balance between damaging and protecting factors on the gastric mucosa: mucin 5B (MUC5B), polypeptide N-acetylgalactosaminyltransferase 8 (GALNT8), cholecystokinin (CCK), claudin 5 (CLDN5), heme oxygenase 1 (HO-1), cyclooxygenase-1 (COX-1) [8,14,30].

Collectively, the results of the studies discussed support the hypothesis that the extent of gastric damage from NSAIDs is determined by the balance between damaging and protective effects within the gastric mucosa. The damaging effects are primarily mediated by COX inhibition, leading to reduced prostaglandin production and its downstream consequences, while the protective effects involve a range of COX-independent mechanisms, as illustrated in Figure 2.

### 3.3. KLS Pharmacokinetics

**Figure 3:**
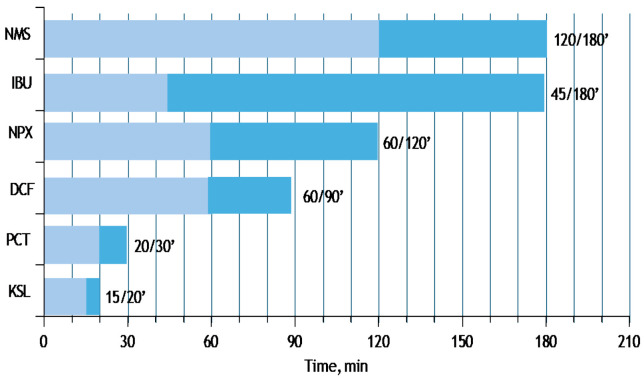
Time to peak concentrations after oral administration of selected NSAIDs and paracetamol (KSL, ketoprofen lysine salt; PCT, paracetamol; DCF, diclofenac sodium; NPX, naproxen sodium; IBU, ibuprofen; NMS, nimesulide). Data from the leaflet of oral formulations.

KLS represents a significant advancement over the parent ketoprofen compound. Through pharmaceutical refinement, the salification process involving the amino acid L-lysine has yielded a novel compound with distinct characteristics: improved efficacy, the ability to use lower therapeutic doses, and enhanced overall tolerability [40]. Following oral administration of standard KA tablets (50–200 mg) in adults, absorption is rapid, with Cmax ranging from 2.6 to 23.0 mg/L occurring between 0.8 and 2.4 h [19]. Compared to traditional ketoprofen, KLS demonstrates increased solubility, and pharmacokinetic analyses have shown that it is absorbed more rapidly and nearly completely, achieving peak plasma concentrations within 15 min, as opposed to 60 min for the original molecule [40]. Lysine salification makes KLS one of the fastest absorbing NSAIDs (Figure 3) [41,42]. KLS exhibits both peripheral and central mechanisms of action [43]. Owing to its high lipophilicity, KLS can cross the blood–brain barrier efficiently, reaching the brain within 15 min post-administration [44,45]. Studies have shown that ketoprofen has higher liposolubility than ibuprofen and other NSAIDs, contributing to this rapid CNS distribution [46]. Furthermore, KLS demonstrates substantial tissue penetration in the upper respiratory tract, making it particularly useful for treating inflammatory conditions such as pharyngitis, otitis, and sinusitis [47]. Its analgesic properties are notably reported to be approximately twice as strong as those of standard ketoprofen. One of the key pharmacological improvements brought about by lysine salification is the ability to lower the defined daily dose (DDD) compared to ketoprofen. This dosage reduction, combined with enhanced solubility and bioavailability, contributes to a better safety and tolerability profile. Specifically, it results in a significant decrease in gastrointestinal side effects, a known limitation of many NSAIDs [15]. Moreover, substantial evidence supports that KLS offers more effective and longer-lasting pain relief, with a quicker onset of action, compared to other NSAIDs. It also demonstrates the highest ratio of anti-inflammatory to analgesic efficacy among comparable agents [16,45]. 

Studies using KA immediate-release capsules established an effect-site rate constant (Ke0) of 0.9 h-1 (95% confidence limits: 0 to 2.1) and a concentration (Ce50) of 0.3 mcg/mL (95% confidence limits: 0.1 to 0.5) for half-maximal pain intensity reduction (PID) [48]. With KLS orodispersible granules, this Ce50 is reached within approximately 6 min for the 40 mg dose and 3 min for the 80 mg sachet, resulting in a correspondingly rapid onset of analgesia [9,49] (Table 2).

## 4. Conclusions

This review highlights the distinct pharmacological advantages of ketoprofen lysine salt (KLS) over ketoprofen acid (KA) and provides valuable insights for clinical practice. Ketoprofen is a potent NSAID with a generally favorable cardiovascular safety profile; however, its use is limited by the risk of gastrointestinal adverse effects. KLS was developed to overcome this limitation, and preclinical studies demonstrate its gastroprotective effects, potentially through a combination of antioxidant mechanisms and the upregulation of key factors involved in maintaining gastric mucosal integrity. In vivo studies further support the improved gastrointestinal and renal tolerability of KLS compared to KA. Furthermore, KLS exhibits a superior pharmacokinetic profile, characterized by increased solubility and faster absorption, with peak plasma concentrations achieved more rapidly than with KA. This translates to a quicker onset of analgesia, a significant clinical advantage for managing acute pain. The enhanced efficacy and tolerability of KLS allow for the potential use of lower doses. These findings suggest that KLS offers a valuable improvement over KA, potentially reducing the risk of gastrointestinal toxicity and providing faster pain relief. This is particularly relevant for patients at risk of NSAID-induced gastrointestinal complications or those requiring rapid analgesia. While preclinical and pharmacokinetic data are promising, further research is needed to fully validate the clinical benefits of KLS. Well-designed clinical trials should directly compare KLS and KA in diverse patient populations, with a focus on rigorously assessing gastrointestinal safety, renal function, cardiovascular safety, and analgesic outcomes. Future studies should also explore the long-term safety profile of KLS and further elucidate the precise molecular mechanisms underlying its gastroprotective effects.

## Figures and Tables

**Table 1:** Association between current use of an individual NSAID and risk of acute myocardial infarction compared with past use of any NSAID (pooled dataset) [24].

NSAIDs	ORpooled	95% Cls
Ketorolac	1.8	1.49 to 2.18
Indometacin	1.51	1.28 to1.80
Etoricoxib	1.39	1.24 to 1.57
Diclofenac	1.28	1.22 to 1.34
Ibuprofen	1.25	1.18 to 1.33
Celecoxib	1.1	1.05 to 1.25
Ketoprofen	1.00	0.86 to 1.16

**Table 2:** Lysine salt PK/PD parameters related to onset of action: ketoprofen lysine salt (KLS); time to maximum concentration (Tmax); concentration that produces half of the maximum PID (Ce50).

	KLS Granules	KLS Sachet
dose mg	40	80
Tmax min	15	15
Ce50 mg/mL	0.3 (Cl 0.1–0.5)	0.3 (Cl 0.1–0.5)
T to Ce50 min	6	3

## Data Availability

No new data were created or analyzed in this study. Data sharing is not applicable to this article.

## Abbreviations

The following abbreviations are used in this manuscript:

4-HNE	4-hydroxy-2-nonenal
CCK	cholecystokinin
Ce50	concentration that produces one-half the maximum PID
CLDN5	claudin 5
Cmax	peak plasma concentrations
COX	cyclooxygenase
CSE	COX-independent cystathionine-γ-lyase
CV	cardiovascular
eNOS	nitric oxide synthase
GALNT8	N-acetylgalactosaminyltransferase
HO-1	heme oxygenase-1
IBU	ibuprofen
KA	ketoprofen acid (KLS)
KLS	ketoprofen lysine salt
LD	linear dichroism
MDA	malondialdehyde
MUC5B	mucin 5B
NSAID	non-steroidal anti-inflammatory drugs
PD	pharmacodynamics
PID	pain intensity reduction
PK	pharmacokinetics
ROS	reactive oxygen species
Tmax	time to max concentration
UGIB	Upper Gastrointestinal Bleeding
